# Comparative Study of Protective Action of Exogenous 2-Cys Peroxiredoxins (Prx1 and Prx2) Under Renal Ischemia-Reperfusion Injury

**DOI:** 10.3390/antiox9080680

**Published:** 2020-07-29

**Authors:** Mars G. Sharapov, Ruslan G. Goncharov, Gleb I. Filkov, Alexander V. Trofimenko, Valery V. Boyarintsev, Vladimir I. Novoselov

**Affiliations:** 1 Laboratory of Mechanisms of Reception, Institute of Cell Biophysics of the Russian Academy of Sciences, PSCBR RAS, 142290 Pushchino, Moscow Region, Russia; goncharov-rg@pbcras.ru (R.G.G.); novoselov-vi@pbcras.ru (V.I.N.); 2 Faculty of Biological and Medical Physics, Laboratory of Special Medical Equipment, Technology and Pharmaceuticals, Moscow Institute of Physics and Technology (MIPT), 141701 Dolgoprudny, Moscow Region, Russia; filkov.gi@mipt.ru (G.I.F.); trofimenko.av@mipt.ru (A.V.T.); boyarintsev.vv@mipt.ru (V.V.B.)

**Keywords:** peroxiredoxin, antioxidant activity, ischemia-reperfusion, kidney, oxidative stress

## Abstract

The pathogenesis of ischemia-reperfusion (I/R) injuries is based on oxidative stress caused by a sharp increase in the concentration of free radicals, reactive oxygen species (ROS) and secondary products of free radical oxidation of biological macromolecules during reperfusion. Application of exogenous antioxidants lowers the level of ROS in the affected tissues, suppresses or adjusts the course of oxidative stress, thereby substantially reducing the severity of I/R injury. We believe that the use of antioxidant enzymes may be the most promising line of effort since they possess higher efficiency than low molecular weight antioxidants. Among antioxidant enzymes, of great interest are peroxiredoxins (Prx1–6) which reduce a wide range of organic and inorganic peroxide substrates. In an animal model of bilateral I/R injury of kidneys (using histological, biochemical, and molecular biological methods) it was shown that intravenous administration of recombinant typical 2-Cys peroxiredoxins (Prx1 and Prx2) effectively reduces the severity of I/R damage, contributing to the normalization of the structural and functional state of the kidneys and an almost 2-fold increase in the survival of experimental animals. The use of recombinant Prx1 or Prx2 can be an efficient approach for the prevention and treatment of renal I/R injury.

## 1. Introduction

It is currently well known that ischemia-reperfusion (I/R) injury is a key factor in the development of many pathological conditions, including various socially significant diseases: myocardial infarction, ischemic stroke, acute renal failure, etc. [1,2,3,4]. Malperfusion (ischemia) leads to a rapid development of pathological processes, including a decrease in ATP level, suppression of aerobic metabolism and switching of the cell to anaerobic processes, which provokes accumulation of lactate, acidification of the cell microenvironment, activation of endogenous oxidases (NADPH oxidase, monoamine oxidase, glycerophosphate dehydrogenase, etc.) [5,6]. All these processes result in deterioration of the structural and functional integrity of ischemic tissues. Restoration of the blood flow (reperfusion stage) in the injured tissue leads to a drastic increase in the level of free radicals, reactive oxygen species (ROS) and reactive nitrogen species (RNS) that damage all biological macromolecules and induce the development of oxidative stress [7,8].

Currently there are several approaches in the treatment of I/R injuries. The first is ischemic preconditioning (IPC), which allows activating proteins and enzymes that increase the tolerance of cells to ROS formed during reperfusion [9]. The use of IPC can be implemented through both physiological (for example, short-term pre-ischemia) and pharmacological (drugs that cause hypoxia, respiratory uncoupling in mitochondria, glycogen synthase kinase (GSK) inhibitors, etc.) manipulations [9,10]. However, the application of IPC is not always possible, since this approach is only effective immediately before I/R, and its use provides no results in the case of developed oxidative stress after I/R. Since the pathogenesis of I/R injuries is associated with oxidative stress, the main direction in the treatment of such pathologies is related to the suppression of free radical processes and growth of ROS/RNS in the affected tissues using antioxidant drugs [11]. Normalization of the level of ROS/RNS contributes to the preservation or more rapid recovery of the damaged tissues. One of the promising approaches in the prevention and treatment of I/R injuries is the use of antioxidant enzymes, which, unlike natural and synthetic low molecular weight antioxidants, are more effective [12].

To suppress oxidative stress, various types of oxidoreductases (both individually and in combination): superoxide dismutase (SOD), catalase (CAT), glutathione peroxidase (GPx) and peroxiredoxins (Prx)—have been tested in in vitro and in vivo experiments with varying degrees of efficiency. It must be noted that some Prx representatives have been investigated previously in models of artificially induced oxidative stress: total irradiation, renal ischemia-reperfusion injury, small intestine, etc. [13,14,15,16,17]. It is the Prx family proteins that have shown high efficiency in oxidative stress neutralization, which is associated with the high antioxidant activity of these enzymes and a wide range of neutralizable hydroperoxides, both organic and inorganic in nature. Mammals have been shown to possess six types of Prx, which are divided into typical 2-Cys (Prx1–4), atypical 2-Cys (Prx5) and 1-Cys (Prx6), according to the number of conserved cysteine residues in the active center and the catalysis mechanisms [18]. Prx1–6 play an important role in maintaining the redox homeostasis in mammals, and their level usually increases in pathologies attended by the development of oxidative stress, thus supporting normalization of the ROS/RNS level in the injured tissues [19,20,21]. Among peroxiredoxins, of particular interest are typical 2-Cys peroxiredoxins (Prx1–4), which, along with the ability to neutralize a wide range of ROS/RNS, act as a chaperone, as well as realize a signaling and regulatory function [22,23].

**Figure 1:**
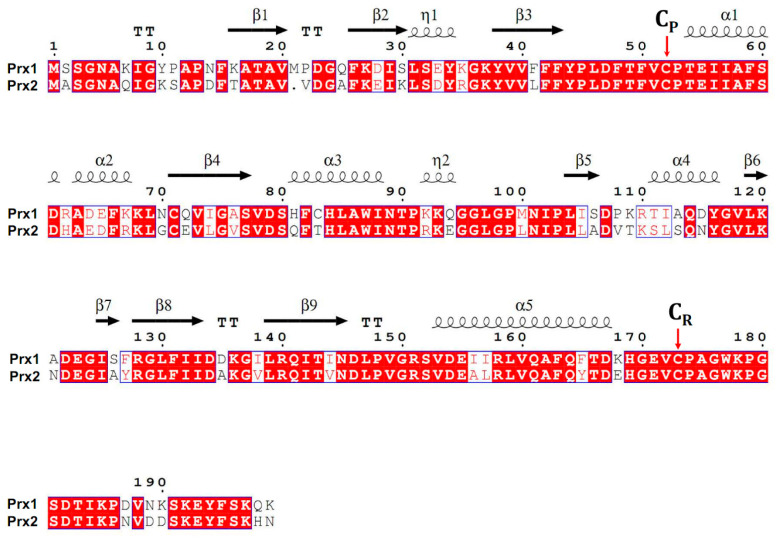
Alignment of amino acid sequences of mouse Prx1 and Prx2 (ClustalOmega, ESPript 3.x). The peroxidatic (C_P_) and resolving cysteine (C_R_) residues indicated by an arrow (Cys52 and Cys173 for Prx1, Cys51 and Cys172 for Prx2). The secondary structure of conserved regions (α-helices, β-strands) is shown above the amino acid sequence of Prx1.

Prx1 and Prx2 are the most common enzymes of the six isoforms of peroxiredoxins; their concentration reaches 0.2% and 1% of the total soluble protein in mammalian cell culture [24]. Prx1 and Prx2 are widely present in all organs and tissues of mammals. It should be mentioned that although Prx1 and Prx2 are highly similar in structure, with a homology of about 90% (Figure 1), they differ in function. Prx1 is more effective as a chaperone (because of Cys83), while Prx2 is more active as a peroxidase [25]. In addition, it has recently been shown that Prx1 and Prx2 differ in sensitivity to peroxidation. Prx2 is more sensitive to peroxidation, while Prx1 retains peroxidase activity in a wider range of H_2_O_2_ concentration [26,27].

Prx1 and Prx2 regulate the content of intracellular peroxides and are involved in intracellular and intercellular signaling [28,29,30,31]. These enzymes play a crucial role in the functioning of blood cells; for instance, *PRDX1* gene knockout leads to hemolytic anemia, while a knockout of the *PRDX2* gene results in damage to erythrocytes and the spleen. At the subcellular level, these enzymes are found in the cytoplasm, nucleus, mitochondria, peroxisomes; moreover, secretory forms have been found in some forms of cancer [18,32]. Mice knockout for the gene *PRDX1* have a shorter lifespan, suffer from hemolytic anemia and, unlike wild-type mice, have a much higher incidence of development of various types of cancer (lymphomas, sarcomas, and carcinomas) over 9 months of age [33]. Mice knockout for *PRDX1* and ApoE demonstrate more extensive atherosclerotic vascular lesions, compared to those which knockout only for the gene of ApoE [34]. Unlike *PRDX1* knockout, a knockout of the *PRDX2* gene does not promote an increase of cancer incidence in animals, but it contributes to the development of hemolytic anemia, due to an important role in the antioxidant defense of erythrocytes, where Prx2 is the third abundant protein after hemoglobin and carbonic anhydrase [35,36]. Prx2 also plays an essential role in maintaining the normal functioning of the cardiovascular system, and the loss of functionally active Prx2 due to gene knockout leads to hemolytic anemia, damage to blood vessels and the spleen. Similarly to the case of *PRDX1*, knockout for *PRDX2* enhances atherosclerosis in ApoE knockout mice [37].

Using a model of retrograde reperfusion of an isolated rat heart (according to the Langendorff method) with physiological solutions containing peroxides, it has been shown that Prx1 and Prx2 have the highest sensitivity to oxidation; thus, they can serve as oxidative stress markers. The oxidation of Prx1 and Prx2 and their conversion to the oligomeric form (with chaperone activity) increased along with an increase in the concentration of peroxides in the perfusion medium [38]. Upon oxidation, Prx1 gets oligomerized (Cys83 plays an important role in this process) and exhibits chaperone function, helping to restore the native structure of RNAs and proteins, both in the cytosol and in the cell nucleus. In erythrocytes, the oligomeric form of Prx2 interacts with the cytoplasmic domain of Band 3 anion transporter, which plays a key role in maintaining the cytoskeleton of erythrocytes, and the oxidized form of hemoglobin, thus preventing its aggregation [39,40,41]. Prx2 is critical for maintaining the structure and function of erythrocytes, as testified by hemolytic anemia and spleen pathologies observed in *PRDX2* knockout mice [35]. In addition, Prx1 and Prx2 interact with numerous transcription factors (NF-κB, HIF-1α, HIF-2α, STAT3, p53, AP-1, c-Myc, p53, etc.), receptors (PDGFR-b) and kinases (ASK1, JNK, p38 MAPKs, etc.), thereby affecting various processes in the cell, including growth, differentiation, and apoptosis [31,42,43,44].

Thus, Prx1 and Prx2 possess peroxidase, chaperone, signaling and regulatory activities, which is of high significance in terms of practical aspect. Therefore, we assume that the use of these enzymes is a promising approach in the prevention and treatment of diseases caused by I/R injuries. In this work, an animal model of renal ischemia-reperfusion injury in mice was used to carry out a comparative study of the protective effect of exogenous Prx1 and Prx2, to provide additional information on the function of these enzymes which are similar in physicochemical characteristics.

## 2. Materials and Methods 

### 2.1. Gene Cloning and Enzyme Production

The cells of mouse bone marrow, where *PRDX1* and *PRDX2* are highly expressed, were used as a source of total RNA. The cells were isolated from extirpated proximal epiphysis of femur with an insulinic syringe by resuspending the cells in 100 µL 10% fetal serum. Total RNA was extracted from the cell suspension using ExtractRNA reagent (Evrogen, Moscow, Russia), following the manufacturer′s recommendations. RNA quality was estimated by electrophoresis in 2% agarose gel in TAE buffer in the presence of ethydium bromide (1 µg/mL). The concentration of RNA was determined using NanoDrop 1000 c spectrophotometer (ThermoFisher Scientific, Wilmington, DE, USA). To prevent possible contamination with genomic DNA, the obtained RNA was treated with RQ1 DNAase (Promega, Madison, WI, USA). For reverse transcription 2 µg total RNA, primer oligo(dT)_15_ and MMLV RT kit (Evrogen, Moscow, Russia) were used. The obtained cDNA was used in PCR with gene specific primers (from Evrogen, Moscow, Russia):

PRDX1m-F: 5′-TAGC**CATATG**TCTTCAGGAAATGCAA-3′, PRDX1m-R: 5′-CCTC**CTCGAG**CTTCTGCTTAGAGAAATACT-3′, PRDX2m-F 5′-CGCA**CATATG**GCCTCCGGCAACGCGCA-3′, PRDX2m-R: 5′-CTA**CTCGAG**GTTGTGTTTGGAGAAGTATTC-3′. (1)

PCR was carried out in a MJMini thermocycler (BioRad, Foster City, CA, USA) using high-fidelity DNA polymerase Encyclo Plus PCR kit (Evrogen, Moscow, Russia). PCR mode: (1) ′′hot start′′ (to exclude nonspecific primer annealing) at 95 °C, 5 min; (2) denaturation at 95 °C, 15 s; (3) primer annealing at 55 °C, 20 s; synthesis at 72 °C, 1 min. Stages (2–4) were repeated 35 cycles. The obtained *PRDX1* and *PRDX2* DNA fragments treated with restriction enzymes NdeI and XhoI (Thermo Scientific, Vilnius, Lithuania) and cloned into pET23b vector following a standard procedure. *E. coli* XL1 Blue cells (Evrogen, Moscow, Russia) were then transformed with the resulting construct. The clones obtained were checked by sequencing (Evrogen, Moscow, Russia). The clones that exhibited 100% coincidence with the sequences of the *PRDX1* and *PRDX2* gene in GenBank were used to derive the protein.

Recombinant proteins contain a His-tag at the carboxyl terminus; therefore, the enzymes were purified under consistent conditions using affinity chromatography on Ni-NTA agarose (Thermo Fisher Scientific, Hilden, Germany), in accordance with the manufacturer′s recommendations. The method for protein extraction and purification is described previously [45]. The purity of the obtained enzymes was not less than 95%, as judged by electrophoresis in 10% SDS-PAGE.

### 2.2. Determination of Peroxidase Activity of Enzymes

The peroxidase activity of Prx1 and Prx2 was estimated according to Kang et al. [46]. The activity was calculated as the quantity (nmol) of H_2_O_2_ or tert-Butyl hydroperoxide (tBOOH) reduced by 1 mg of enzyme over 1 min. The peroxidase activity of recombinant Prx1 was 600 and 450 nmol/min/mg against H_2_O_2_ and t-BOOH, respectively. For Prx2, the peroxidase activity was 350 and 200 nmol/min/mg for H_2_O_2_ and t-BOOH, respectively.

### 2.3. Determination of Thermal Stability of Enzymes

Solutions of Prx1 or Prx2 (at a concentration of 1 mg/mL) were heated in a BioRad MJMini thermocycler, with the temperature gradient set in the range from 37 °C up to 90 °C (the deviation of temperature from the set values 0.2 °C). The volume of samples was 100 µL. Heating was carried out for 30 min. Residual peroxidase activity of the enzymes was determined at 37 °C. Prx1 and Prx2 are quite similar in thermal stability which correlates with chaperone activity [15]. Both enzymes possess 100% peroxidase activity in a wide temperature range of 37–58 °C, retain at least 50% activity at 64 °C and nearly completely lose the activity when heated above 70 °C.

### 2.4. Determination of Endotoxins Level

Determination of the level of bacterial endotoxins in the recombinant Prx1 and Prx2 proteins was carried out using the LAL test, semi-quantitative gel-thrombus method, in accordance with the manufacturer′s recommendation (Sigma-Aldrich, Saint Louis, MO, USA). As a positive control, an LPS preparation from *E. coli* 055:B6 (Sigma-Aldrich, Saint Louis, MO, USA) cells was used. The E-TOXATE reagent from *Limulus polyphemus* (Sigma-Aldrich, Saint Louis, MO, USA) was used as the LAL reagent. The sensitivity of the LAL reagent was 0.05 EU/mL. The LPS content in the Prdx1 and Prx2 preparations was about 3.5 EU/mL or 3.5 ng LPS per 1 mg of Prdx1 and Prdx2 proteins.

### 2.5. Animals

The studies were carried out in *BALB/c* mice (8 weeks old, weight 25–30 g) (vivarium of ICB RAS, Pushchino). The experiment protocol was approved by the institutional Ethics Committee of the Institute of Cell Biophysics RAS and all experiments were carried out according to international regulations listed in the European Convention for the Protection of Vertebrate Animals used for Experimental and Other Scientific Purposes (ETS 123) and ICB RAS Manual for Working with Laboratory Animals №57 (30.12.2011), ethical protocol №2019/5. The animals had *ad libitum* access to food and water, but the access to food was restricted 24 h before surgery.

### 2.6. Animal Model of Renal Ischemia-Reperfusion Injury

The animals were anesthetized by intramuscular injection of Zoletil-100 (Virbac Sante Animale, Carros, France) and Rometar-20 (Bioveta, Komenského Ivanovice na Hané, Czech Republic) in 0.9% NaCl solution (40 μg and 7 μg per 1 g of body weight, respectively). The duration of anesthesia was 1.5–2 h. Operations in animals were carried out according to the procedure described in the work [47], with minor modifications. After the beginning of anesthesia, small lateral incisions of skin and muscular layers on both sides were made, opening the access to renal arteries and veins. Then the left and right renal arteries and veins were clamped simultaneously, which caused the blockage of blood flow to and from the renal tissues, i.e., ischemia. A visible sign of ischemia onset was a change in the kidney color from pale pink to deep purple. The duration of ischemia was 30 min, after that the clamps were removed for restoration of circulation in the tissue (reperfusion stage). The beginning of reperfusion was attended by a change of the kidney color from deep purple to pale red. The lateral incisions were sutured and washed with an antiseptic liquid. After the operation, the animals were provided with food and water *ad libitum*. 24 h and 72 h after the operation the animals were killed by decapitation and the kidneys were excised.

To test the therapeutic effect of exogenous Prx1 and Prx2 proteins, the solution of the corresponding recombinant protein was injected into the tail vein to a final concentration of 20 μg/g of bodyweight 15 min before the beginning of ischemia. Selection of the injection method and the concentrations of protein solutions was carried out according to previous studies [13,14].

### 2.7. Histological Analysis

The kidney tissues were fixed in 10% formaldehyde solution, followed by dehydration of samples in an increasing gradient of ethanol concentration and enclosure into paraffin. Three-micron paraffin sections were prepared on a microtome Microm HM355S (Thermo Fisher Scientific, Walldorf, Germany). The obtained sections were stained with hematoxylin and eosin (Biovitrum, Saint-Petersburg, Russia). Histological analysis was carried out on a Leica DM6000 microscope (Leica, Wetzlar, Germany). Typically, 15–20 fields were inspected for each section of 3 different slides, at 200–500-fold magnification.

### 2.8. Electrophoresis and Immunoblotting

To estimate the circulation time of exogenous Prx1 and Prx2 in the animal blood, 1 mg Prx1 or Prx2 was introduced intravenously into three male *BALB/c* mice and the blood was subsequently sampled (−50 µL) after 0.25, 1, 2, 4, 6 and 24 h for analysis of changes in the Prx1 or Prx2 content. Exogenous recombinant Prx1 and Prx2 contain a His-tag on their C-termini, which enables them to specifically trace the presence of the recombinant proteins. To determine changes in caspase-3 levels in kidney tissues, about 40 mg of the tissue was collected. Kidney and serum protein samples were separated by electrophoresis in 10% SDS-PAGE using a Mini Vertical Unit SE 250 (GE Healthcare, Chicago, IL, USA) and transferred on to a PVDF membrane Amersham Hybond P (GE Healthcare, Chicago, IL, USA) using a TRANS-BLOT SD semidry transfer unit (Bio-Rad, Hercules, CA, USA). The following primary antibodies were used: monoclonal rabbit anti-His antibodies (1:1000, #12698, Cell Signaling technology, USA); rabbit monoclonal antibody for Caspase 3 (1:1000, 9H19L2, Thermo Fisher Scientific, Rockford, IL, USA); rabbit antibody for *β*-Actin (1:1000, #4967, Cell Signaling, Danvers, MA, USA). Secondary goat antibodies against rabbit immunoglobulins, conjugated with horseradish peroxidase (1:1000, *p*-GAR Iss, IMTEK, Moscow, Russia), were used for immunoblotting in accordance with the manufacturers’ recommendations. The detection was carried out using diaminobenzidine, DAB (Amresco, USA). Densitometry was implemented using ImageJ software v.1.50i (www.imagej.nih.gov). Data were normalized to *β*-Actin.

To assess the changes in the activity of Prx1 and Prx2 proteins after incubation in the blood, animals (3 mice for each time point) were injected intravenously with 1 mg of the corresponding protein. Then after 10, 60 or 120 min the animals were killed by decapitation with subsequent blood sampling. Erythrocytes were removed by centrifugation (1500× *g*, NT). The resulting plasma was 10-fold diluted with buffer (1xPBS, 10 mM imidazole, pH 7.4) for application to a Ni-NTA agarose column and the protein was purified according to [45]. After that, the residual peroxidase activity of Prx1 and Prx2 was assessed according to the previously described technique [46].

### 2.9. Gene Expression Level Analysis

Gene expression level was determined by reverse transcription and real-time PCR. Total RNA was isolated from renal tissue samples with ExtractRNA reagent (Evrogen, Russia). RNA quality was estimated electrophoretically in 2% agarose gel. RNA concentration was determined using NanoDrop 1000 c spectrophotometer (USA). Two micrograms of total RNA was used per reverse transcription reaction with MMLV reverse transcriptase and standard dT_15_ oligonucleotide (Evrogen, Moscow, Russia). The synthesized cDNA was used for real-time PCR with 200 nm gene-specific primers (Table 1). Real-time PCR was carried out using DNA amplifier DTlite (DNA-Technology, Moscow, Russia) with qPCRmix-HS SYBR kit (Evrogen, Moscow, Russia). The PCR cycling mode was as follows: (1) «hot-start»: 95 °C, 5 min; (2) denaturation, 95 °C, 15 s; (3) primer annealing and DNA synthesis at 60 °C, 30 s. Stages (2) and (3) were repeated 40 times. The threshold cycle (Ct) value was determined using DTmaster software (DNA-technology, Moscow, Russia). The signal was normalized to that obtained for the gene of β-Actin (Actb). ∆Ct value was calculated by the formula ∆Ct = Ct (gene of interest) – Ct (Actb); ∆∆Ct was calculated ∆Ct (control) − ∆Ct (experiment). The 2^-∆∆Ct method was used to calculate differences in genes expression [48].

### 2.10. Determination of MDA Level

The level of malonic dialdehyde (MDA) was determined by a standard technique with thiobarbituric acid (TBA). To a 20–30 mg tissue sample, 450 μL of 1% H_3_PO_4_ and 150 μL of 0.8% TBA were added, and the mass was homogenized with a teflon pestle. Then the mixture was heated on a boiling water bath for 45 min. After cooling, 380 μL of *n*-butanol was added and mixed thoroughly. The layer of *n*-butanol was separated by centrifugation. The optical density of water phase was measured at 546 nm with a Multiskan instrument (Labsystem Plus, Helsinki, Finland).

### 2.11. Biochemical Blood Analysis

Blood samples were collected from the animals of control and experimental groups before I/R injury, 24 h and 72 h after I/R injury of the kidneys, according to Gowda et al. [49]. Biochemical blood analysis was performed using a biochemical express analyzer Reflotron Plus (Roche Diagnostics, Rotkreuz, Switzerland) according to the manufacturer′s instructions.

### 2.12. Statistical Data Analysis

Statistical data analysis was carried out in SigmaPlot 11 software package (Systat Software Inc, San Jose, CA, USA). Statistical significance between experimental groups was determined using unpaired Student′s *t*-test and one-way ANOVA analysis. *p* < 0.05 was considered statistically significant. The results were presented as mean value ± standard deviation (SD).

## 3. Results

### 3.1. Estimation of the Circulation Time of Prx1 and Prx2 in the Bloodstream of Animals

**Figure 2:**
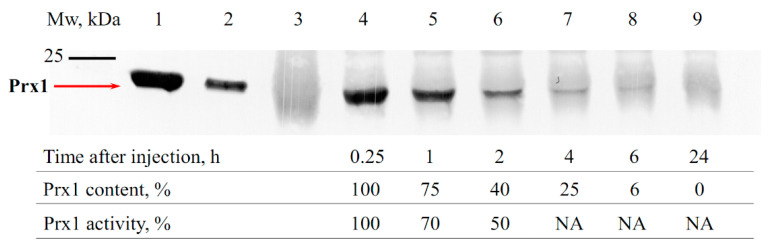
Immunoblotting of mouse blood serum after intravenous Prx1 injection. 1,2—purified recombinant Prx1 (500 and 100 ng, respectively); 3—blood plasma of control animals which did not receive Prx1; 4–8—blood serum samples of mice 0.25, 1, 2, 4, 6 and 24 h after intravenous injection of 1 mg of Prx1.

**Figure 3:**
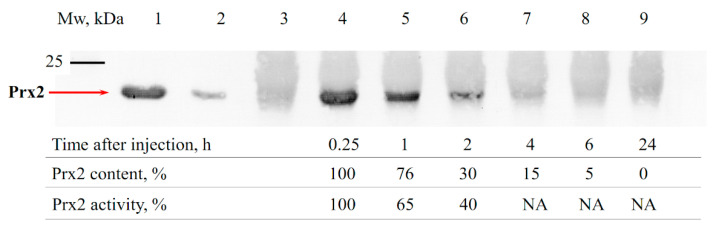
Immunoblotting of mouse blood serum after intravenous Prx2 injection. 1,2—purified recombinant Prx2 (500 and 100 ng, respectively); 3—blood plasma of control animals which did not receive Prx2; 4–8—blood serum samples of mice 0.25, 1, 2, 4, 6 and 24 h after intravenous injection of 1 mg of Prx2.

Recombinant mouse Prx1 and Prx2 are close in physicochemical properties, in particular, having similar thermal stability. However, Prx1 has a higher (nearly 2-fold) peroxidase activity against both inorganic peroxide H_2_O_2_ and organic tert-butyl hydroperoxide (tBOOH), which indicates a potentially higher antioxidant activity of Prx1 compared to Prx2 (see Materials and Methods section). In addition to the antioxidant activity, the time (duration) of the presence of exogenous Prx1 and Prx2 in the kidneys during I/R damage plays an important role in their protective effect. Using immunoblotting, the alteration in the amount of the recombinant Prx1 and Prx2 in the blood serum of animals was analyzed in the early period following protein administration (a 15 min interval was chosen, which corresponds to the beginning of ischemia, according to a previously described animal model), as well as after a long time (6 h) following intravenous introduction (Figure 2 and Figure 3).

As seen from Figure 2 and Figure 3, about 75% of the initial amount of the introduced enzymes was present in the animal blood during the first hour after intravenous injection of Prx1 and Prx2. Over time, the amount of exogenous enzymes Prx1 and Prx2 in the animal blood serum decreased, showing an approximately 4–6-fold reduction after 4 h. Notably, the level of Prx1 after injection into the bloodstream remained at a higher level compared to Prx2, which can affect the lifetime of the protein in the blood and the ability to neutralize ROS/RNS after I/R injury. Thus, exogenous recombinant proteins Prx1 and Prx2 were present in the blood of animals during the entire ischemic period (30 min) at a rate of at least 75%, and at least 30–40% of the protein was present in the blood during the first two hours upon subsequent reperfusion. The decrease in the level of exogenous Prx1 and Prx2 in the bloodstream may be due to their distribution in the tissues. For instance, Prx6 and its modified forms have been shown to distribute under the vascular endothelium, at the border with the basement membrane [50]. It should be mentioned that in addition to the reduction of the Prx1 and Prx2 concentration in the blood of animals after administration, a decrease in the peroxidase activity of exogenous enzymes over time was also observed. For example, purification of Prx2 protein from animal blood plasma using affinity chromatography (IMAC) and subsequent evaluation of protein peroxidase activity testified that after 60 min and 120 min of incubation the Prx2 activity decreases by about 40% and 60%, respectively. Thus, exogenous peroxiredoxins (Prx1 and Prx2) circulating in the blood get oxidized (probably irreversibly) over time, which reduces their antioxidant activity and nephroprotective effect.

### 3.2. Survival of Animals after I/R and with Prior Administration of Prx1 and Prx2

**Figure 4:**
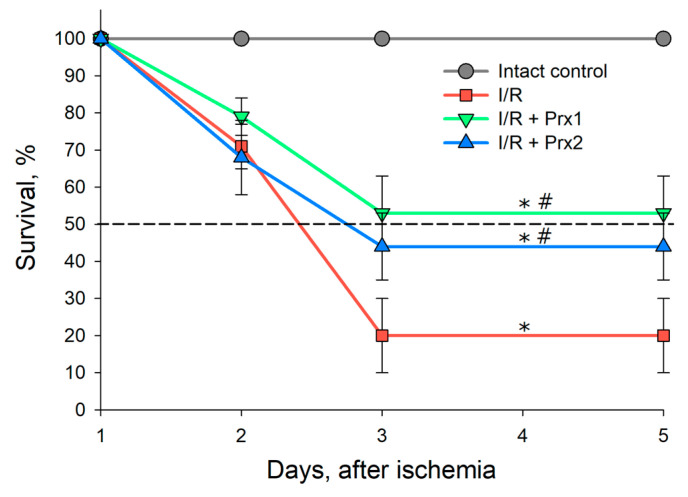
Survival of mice during 5 days after 30 min ischemia of both kidneys and subsequent reperfusion, and after injection of recombinant enzymes, Prx1 and Prx2, 15 min before ischemia (*n* = 30 for each group). *p* ˂ 0.05 relative to the * intact control and # I/R.

At the first stage of the study, the survival of animals was estimated over 5 days after I/R injury of both kidneys and preliminary administration of the recombinant enzymes Prx1 and Prx2 15 min prior to a 30 min ischemia (Figure 4).

After 72–120 h, about 20% of animals survived in the group with I/R without prior administration of recombinant enzymes, as seen from Figure 4. In the groups with preliminary injection of Prx1 and Prx2, the survival rate over the same period was 53% and 44%, respectively. Thus, the first 72 h after I/R injury of both kidneys is the critical period for the survival of animals.

It must be noted that changes in the mass of the animal kidneys were registered after I/R and prior injection of recombinant Prx1 and Prx2 (Table 2). From Table 2 it is seen that after 24 h animals in the group with I/R exhibited a 1.5-fold increase in the kidney mass, which may be due to acute tissue edema [51]. In the groups with prior introduction of Prx1 and Prx2 prior to I/R, a similar increase in the mass of the kidneys was observed—by 1.3 and 1.4 times, respectively. Following 72 h, normalization of kidney mass was observed in all the groups, with a slight decrease of mass in the control group I/R.

### 3.3. Histological Analysis of Kidney Tissue after I/R Injury and with Prior Administration of Prx1 and Prx2

**Figure 5:**
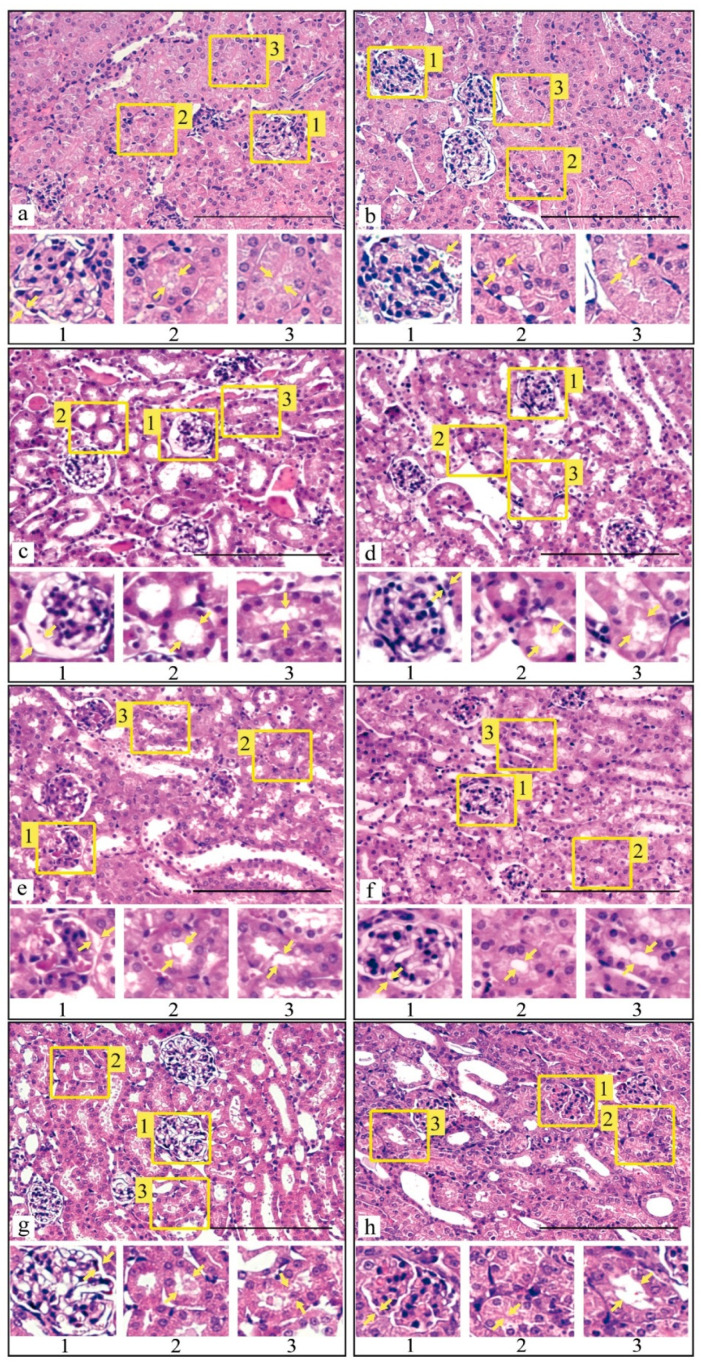
Structure of the cortical layer of mouse kidney after injection of recombinant peroxiredoxins followed by ischemia-reperfusion. 1—Glomerulus, 2—Distal tubules, 3—Proximal tubules. Arrows indicate areas of morphological changes. Intact control—(**a**) and (**b**). The duration of ischemia was 30 min, the duration of reperfusion—24 h (**c**) and 72 h (**d**), without treatment. After intravenous injection of Prx1 15 min prior to 30-min ischemia, the duration of reperfusion was 24 h (**e**) and 72 h (**f**). After intravenous injection of Prx2 15 min prior to 30-min ischemia, the duration of reperfusion was 24 h (**g**) and 72 h (**h**). Staining: hematoxylin-eosin. Magnification: 500×. Scale: 200 µm.

From literature it is known that renal function persists within normal limits, provided that at least 50% of nephrons are preserved [52]. To clarify, what morphological changes in the renal tissue occur in I/R alone and with prior administration (15 min before) of recombinant enzymes Prx1 and Prx2 before 30 min of ischemia and subsequent reperfusion (24 and 72 h), a histological analysis was performed (Figure 5).

Morphological analysis of histological sections showed that 30-min ischemia and subsequent 24 h reperfusion of mouse kidneys leads to congestion of the interstitium and glomerular capillary loops in the control group. In the epithelium of the renal tubules, especially in convoluted tubules, pronounced dystrophic damage was revealed, mainly hydropic degeneration, with destruction of the apical regions of epithelial cells. In some convoluted tubules, necrosis and desquamation of tubular epithelial cells were registered. The lumen of the tubules was filled with protein detritus (Figure 5c). Following 72 h after blood flow restoration in the kidneys, moderate congestion of the glomerular capillary loops persisted. In the epithelium of the convoluted tubules, ballooning degeneration of the cells with destruction of their apical regions was identified. A pronounced eosinophilia of the cytoplasm of the epithelial cells was probably related to massive protein denaturation. Widened lumens of the convoluted tubules of the kidneys were due to destructive changes in the epithelial cells (Figure 5d). Dystrophic changes, but without cell destruction, were also found in the epithelium of the straight tubules.

Prx1 injection 15 min prior to 30-min ischemia and subsequent 24-h reperfusion reduced congestion of the interstitium and glomerular capillary loops. The integrity of the glomeruli of the nephrons was not violated; the size increased inconsiderably. Dystrophic alterations in the epithelium of the renal convoluted and straight tubules, characterized by the presence of hydropic dystrophy with minor destruction of the apical regions of epithelial cells, were expressed marginally. The lumens of the convoluted tubules appeared to be widened; some of them demonstrated the presence of protein detritus, as well as desquamated tubular epithelial cells (Figure 5e). Following 72 h after renal blood flow restoration, moderate blood filling of the glomerular capillary loops retained. Minimal dystrophy was observed for the convoluted and straight tubules. The convoluted tubule epithelium was characterized by poorly expressed hydropic and protein dystrophy. The lumens of the convoluted tubules were slightly widened, with preserved desquamation of epithelial cells. The destruction of epithelial cells was minimal. The presence of protein fluid and tissue detritus in the lumens of the convoluted tubules also appeared to be minimal (Figure 5f).

Injection of Prx2 15 min prior to 30-min ischemia and subsequent 24-h reperfusion caused congestion of the interstitium and glomerular capillary loops. The integrity of the glomeruli of the nephrons was not violated, although they were found to be hypertrophic. In the epithelium of the renal tubules, especially in convoluted tubules, pronounced dystrophic damage was revealed, mainly hydropic degeneration, with destruction of the apical regions of epithelial cells. In some convoluted tubules, desquamation of epithelial cells was found. The lumens of the tubules were widened, with the presence of protein fluid and tissue detritus (Figure 5g). After 72 h of reperfusion, congestion of capillary loops and destructive changes in the convoluted and straight tubules were preserved in the kidney tissue. The lumens of the convoluted tubules were widened and exhibited no tendency to further widening. Accumulation of protein fluid containing tissue detritus and desquamated epithelial cells was revealed in the lumens. However, the intensity of the above changes manifested on the first and third days is lower than that in the group without prior administration of the recombinant enzyme (Figure 5h). The overall results of the histological analysis of renal tissue after I/R and with prior injection of the Prx1 and Prx2 enzymes before I/R are presented in Table 3.

Based on the histological analysis of the renal cortex following 24 and 72 h after I/R, it can be concluded that the use of recombinant enzymes Prx1 and Prx2 15 min before 30-min ischemia contributes to a significant reduction in renal tissue damage. At the same time, it is worth noting that, according to a number of morphometric characteristics, Prx1 is more effective compared to Prx2.

### 3.4. Biochemical Analysis of Animal Blood after I/R Injury and Prior Administration of Prx1 and Prx2

It is well known that the blood level of creatinine and urea reflects the physiological state of the kidneys. The content of urea (Table 4) and creatinine (Table 5) in the blood of animals after I/R and with preliminary administration of Prx1 and Prx2 prior to I/R was assessed on the first, second and third days after I/R injury.

Following 24 h after I/R injury, an approximately 5-fold increase in the urea concentration and more than 6-fold increase of creatinine was observed in the blood of animals, as compared to the intact group (Table 4 and Table 5). After 24 h following I/R, the group with preliminary administration of Prx2 demonstrated a near 4 times higher concentration of urea and more than 5 times higher creatinine in the blood, in comparison to the physiological norm. The concentrations of urea and creatinine were the highest: 3-fold increase for urea and 4-fold increase for creatinine. Following 48 h after I/R, all groups exhibited a marked 1.5–2-fold decrease in the concentration of urea and creatinine in the blood of experimental animals. After 72 h, the urea level in all groups was stabilized at a value slightly above the physiological norm. The creatinine content in the groups with I/R and prior administration of Prx1 and Prx2 was 2–2.5 times higher than the physiological norm.

Based on the data obtained, it can be concluded that under conditions of I/R injury of both kidneys, prior administration of Prx1 or Prx2 (15 min prior to 30-min ischemia and subsequent reperfusion) can effectively maintain renal excretory function. At the same time, the efficiency of Prx1 application is higher compared to Prx2.

### 3.5. MDA Level in the Renal Tissue after I/R and with Prior Administration of Prx1 and Prx2

**Figure 6:**
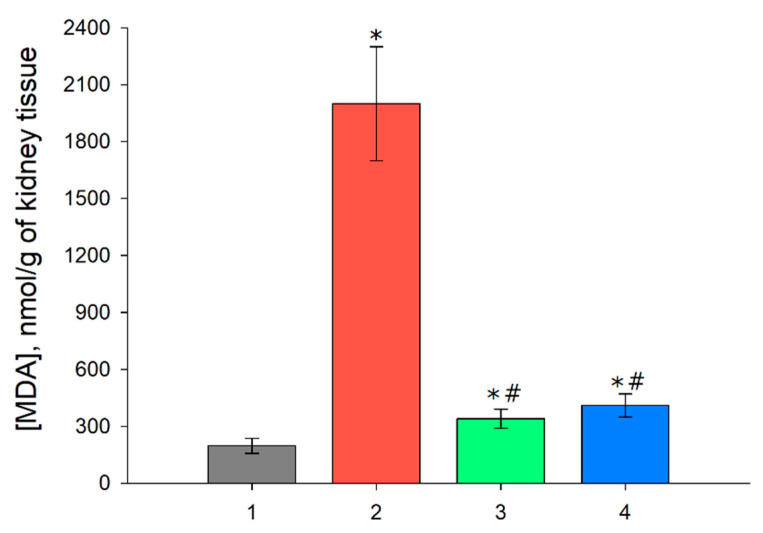
MDA level in kidney tissues (*n* = 5 for each group). 1—Intact mice; 2—24 h after ischemia-reperfusion; 24 h after ischemia-reperfusion injury preceded by injection of Prx1 (3) and Prx2 (4). *p* ˂ 0.05 relative to the * intact control and # I/R.

Since I/R of organs is attended by oxidative stress in the damaged tissues, the level of lipid peroxidation increases, with malondialdehyde (MDA) being one of the end products of this process. An analysis of the MDA content in the kidney tissue of animals was performed following one day after I/R injury and with preliminary administration of Prx1 and Prx2 before I/R (Figure 6).

As seen from the obtained data on Figure 6, there was approximately an 10 times increase in the content of MDA in the renal tissue following 24 h after the I/R injury. The preliminary application of Prx1 and Prx2 significantly reduced the amount of MDA, by more than 5 times compared to the group without prior injection of exogenous enzymes, indicating a decrease in the process of lipid peroxidation and, consequently, oxidative stress.

### 3.6. Assessment of Gene Expression in the Kidney Tissue after I/R Injury and with Prior Administration of Prx1 and Prx2

To understand the molecular mechanisms of the protective effect of exogenous Prx1 and Prx2, the changes in the expression level of some marker genes were estimated (Table 1). Table 6 presents data on the level of expression of certain marker genes in the kidney tissue following one day after I/R injury and with prior administration of Prx1 and Prx2 before I/R. Notably, 48–72 h after I/R, the gene expression was normalized, approaching the values of the intact animals (data not shown).

Kidney injury molecule-1 (KIM-1) is a recognized sensitive marker of acute renal injury [53]. Indeed, the increase in the KIM-1 expression was more than 100-fold in the groups with I/R injury. The groups with prior administration of Prx1 and Prx2 exhibited a 70–80-fold increase in the expression level of KIM-1, which is 1.2–1.5 times lower compared to the control I/R injury group, testifying the protective role of exogenous Prx1 and Prx2 in renal I/R injury.

Transcription factor NRF2 is the main transcription factor that regulates the expression level of antioxidant response genes, thereby playing a key role in maintaining tissue redox homeostasis. In renal I/R injury, a significant increase in the expression of NRF2 (7.5-fold) was observed in the control mice, and a slightly lower induction of NRF2 (4-fold) was registered in the group with Prx2 injection prior to I/R. Despite the significant rise in the NRF2 level in these groups, we failed to detect a substantial (more than 2–3-fold) increase in the expression of genes encoding antioxidant enzymes, with the exception of the genes for SOD2, PRDX1, PRDX2 and PRDX6, for which the expression level altered 5–6-fold after 24 h following I/R (Table 1). Preliminary administration of Prx1 and Prx2 prior to I/R injury normalized the expression of NRF2 and antioxidant response genes almost to the level of the intact animals.

The expression level of transcription factor NF-kB in I/R injury was 5-fold increased, which probably reflects an adaptive reaction to the development of oxidative stress. Against the background of increased values for NF-kB, a noticeable augmentation of the expression of the IL-6 (2.5-fold) and IL-18 (7-fold) genes was revealed, which indicates the development of the inflammatory process and stimulation of the immune response. In the group with prior injection of Prx2, a significant increase was observed in the expression of NF-kB (4-fold) and IL-6 (5-fold) in comparison with the intact group. It should be noted that IL-6 can not only have a pro-inflammatory effect, but is also able to stimulate regenerative processes in the cell [54]. Preliminary injection of Prx1 reduced the expression level of NF-kB and IL-6 by approximately 2–3 times in comparison with the previous groups. This effect may be possibly due to the fact that Prx1 injection results in a more effective decrease in the ROS level and normalization of homeostasis in the cells under the conditions of this pathology. Injection of Prx1 and Prx2 before I/R was attended by a significant decrease in the expression of the pro-inflammatory cytokine IL-18 (7-fold) in comparison to the control I/R group.

The expression of nitric oxide synthase genes (inducible iNOS and endothelial eNOS) in I/R injury was substantially enhanced: 14-fold and 22-fold for iNOS and eNOS, respectively, leading to an increase in the level of NO in the blood, which is apparently an adaptive response aimed to restore the blood circulation. Upon prior administration of Prx1 and Prx2, such a sharp rise in iNOS and eNOS was not registered, although their level was 2–4 times higher relative to the control group.

The obtained data indicate a 4–7-fold augmentation in the expression level of the transcription factor AP-1 (regulating cell apoptosis) in the I/R group and in the group with preliminary administration of Prx2 prior to I/R. The same groups, along with the increased level of AP-1, exhibit a 3.5–4.5-fold rise in the level of effector caspase-3 (Casp-3). This increase may be due to enhanced apoptotic cell death in the renal tissue. In the group with prior administration of Prx1, the expression of AP-1 and Casp-3 was significantly reduced, being close in the values to the intact animals, which indicates a reduction in apoptotic cell death.

**Figure 7:**
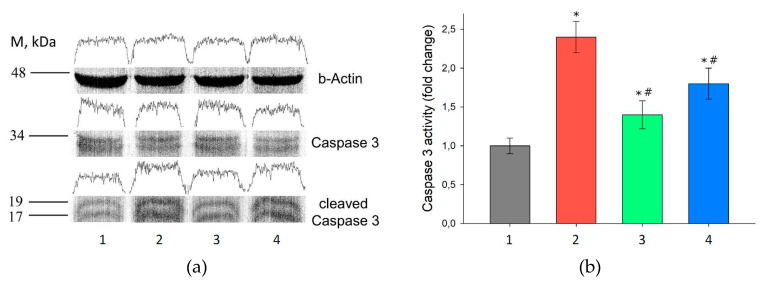
Immunoblotting of kidney tissue for beta Actin and Caspase-3 (*n* = 5 for each group). (**a**) WB: 1—Intact mice; 2–24 h after ischemia-reperfusion; 24 h after ischemia-reperfusion injury preceded by injection of Prdx1 (3) and Prx2 (4). Data normalized to beta Actin (45 kDa). (**b**) The level of activation of Caspase-3 was determined by the ratio of pro-Caspase-3 (35 kDa) to cleaved Caspase-3 (17–19 kDa). *p* ˂ 0.05 relative to the * intact control and # I/R.

To verify that the increase in the level of the Casp-3 expression is indeed associated with its induction in the renal tissue cells and the activation of apoptosis in I/R injury, immunoblotting of the kidney tissues was performed following 24 h after I/R and with preliminary injection of Prx1 and Prx2 enzymes before I/R (Figure 7).

As seen from Figure 7, the groups with I/R and with prior Prx2 injection had a 1.8–2.4-fold caspase-3 activation compared to the intact group. This observation might indicate an increase in apoptotic cell death in the renal tissue in these experimental groups. Suppression of caspase-3 activation and a 1.25-fold growth in the level of its active form was observed in the group with preliminary injection of Prx1 prior to I/R.

## 4. Discussion

The data presented testified that preliminary injection of recombinant Prx1 or Prx2 prior to I/R injury significantly reduces the severity of kidney damage. This effect is manifested through a reduction in animal mortality (Figure 4), a substantial preservation of both the morphology (Figure 5, Table 3) and the filtration capacity of the kidneys (in relation to creatinine and urea) (Table 4 and Table 5). According to all criteria under consideration, Prx1 is approximately 15–20% more effective than Prx2, which may be due to its higher enzymatic activity (see Materials and Methods section), because the peroxidase activity of Prx1 in vitro is about 20–30% higher than that of Prx2. In addition, the recombinant Prx1 circulates longer in the blood of animals, which increases the elimination time of ROS/RNS in the blood. The findings of the present study coincide with the data on the nephroprotective role of another representative of the peroxiredoxin family—Prx6 in the protection of the kidneys in I/R injury. Interestingly, Prx1 and Prx6 are comparable in efficiency, despite the significantly lower peroxidase activity of Prx6, which can be explained by different substrate specificity of these enzymes, i.e., notwithstanding the lower peroxidase activity, Prx6 is able to neutralize peroxide substrates that cannon be eliminated by Prx1, for example, phospholipid peroxides [16]. The combined use of Prx1 and Prx6 may be a more promising approach in preventing the after-effects of I/R injuries and preserving ischemic organs during transplantation.

In I/R injury, a compensatory rise in the expression level of endogenous antioxidant enzymes takes place in response to an increase in the level of ROS/RNS [55]. I/R injury is accompanied by alterations in the redox homeostasis of the cells, which results in the activation of intracellular redox-sensitive transcription factors, such as NRF2 [56]. The activation of the transcription factor Nrf2 under renal I/R injury indicates an increase in the expression level of genes encoding antioxidant enzymes due to interaction with the cis-regulatory element ARE (antioxidant response element). In particular, an important protective role has been shown for endogenous peroxiredoxins in the neutralization of oxidative stress in the kidneys upon I/R injury. The level of peroxiredoxins increases in I/R, and the expression profile in the nephron of the kidney demonstrates a segment-specific pattern [55]. In response to I/R, the level of Prx1 increases in the Henle’s loop, Prx2—in the Shumlyansky-Bowman capsule, Prx3—in the proximal and distal convoluted tubules, Prx4 does not show significant changes in the expression level, an increase in the level of Prx5 is observed in all tubules, whereas Prx6 increases in the proximal convoluted tubule and in the Henle’s loop. Such segment-specific expression of Prx16—may be associated with the production of different types of hydroperoxides in various segments of the nephron in I/R. Peroxiredoxins are known to differ in the efficiency of neutralization of various types of hydroperoxides, and some Prxs are able to neutralize only a certain type of peroxide [57]. For instance, among mammalian peroxiredoxins, Prx2, Prx5 and Prx6 can reduce peroxynitrite, while phospholipids hydroperoxides can be reduced only by Prx6. As mentioned earlier, typical 2-Cys peroxiredoxins (Prx1–4) exhibit chaperone activity under oxidative stress conditions, which may be of importance in the regeneration of damaged nephron areas upon renal I/R injury [58,59,60]. In our experiments the most significant changes in the expression in the renal tissue were observed for Prx1, Prx2, and Prx6, which may be related to the highest prevalence of these isoforms of peroxiredoxins in the cells, as well as their important physiological role in maintaining the redox homeostasis. Using an animal model, it was shown that in mice with prior administration of exogenous Prx1 or Prx2 before bilateral renal I/R, a decrease in the level of NRF2 expression takes place in the damages tissues, as well as a decrease in the expression of some antioxidant response genes regulated by this transcription factor (Table 6). This fact may indicate normalization of the redox homeostasis in the kidney tissue upon I/R injury, under the action of recombinant Prx1 and Prx2.

One of the key transcription factors, the activation of which helps to maintain the normal cell homeostasis under stress conditions and, ultimately, controls the balance between the survival and death of cells, is the transcription factor NF-kB [61]. The expression level of the NF-kB-coding gene in the group of animals with I/R injury without treatment was 5–10-fold increased, aiming to the activation of pro-inflammatory and reparative processes. It has been shown that NF-kB interacts with c-Jun N-terminal kinase (JNK) and thereby reduces its activity, preventing necrotic and apoptotic cell death [62,63,64]. Since NF-kB is a universal transcription factor, its activation can regulate the expression of a wide variety of genes, including different interleukins [62,65]. Against a background of increased NF-kB, a marked rise in the level of the expression of the IL-6 and IL-18 genes was registered in the same groups. An increase in IL-6 and IL-18 may be due to stimulation of the cell immune response and modulation of the activity of Th1 cells, cytotoxic T-lymphocytes, NK cells, macrophages and dendritic cells [54,65]. Prior injection of Prx1 or Prx2 reduces the expression level of NF-kB and IL-18. This may result from a reduction of the ROS level and normalization of cell homeostasis under the action of exogenous Prx1 or Prx2, as in the case of the use of some other antioxidants [66,67,68,69]. Despite the decrease in the levels of NF-kB and IL-18, these groups exhibit a significant increase (2–8-fold) in the activation of the IL-6 level compared to the control group, which may be explained by the fact that IL-6 can have not only a pro-inflammatory effect via the trans-signaling mechanism, but also an anti-inflammatory effect capable of activating regenerative reactions in the cell [54]. It must be mentioned that Prx1 and Prx2 affect the level of NF-kB indirectly through stimulation of the TLR4/NF-kB signaling pathway. In most types of cells peroxiredoxins have intracellular localization (with the exception of certain secretory forms) [70] and function as danger/damage signaling molecules—DAMPs (Damage-Associated Molecular Patterns)—when entering into the extracellular space [71]. DAMPs inform the organism of potential danger through interaction with receptors on the cell surface (NLRs, RLRs, ALRs, TLRs), thereby stimulating the immune system and activating regeneration processes [71,72]. Previously, it has been shown that Prx1, Prx2, Prx5 and Prx6 are potent DAMPs in ischemic stroke, with TLR4 being their main recognition receptor. Through interaction with the TLR4 receptor, peroxiredoxins (Prx1, Prx2, Prx5 and Prx6) express pro-inflammatory/regenerative factors via the TLR4/MyD88/NF-kB signaling pathway [14,73,74,75,76,77,78,79]. Moreover, we suggest that intravenous injection of recombinant Prx1 or Prx2 in animals before renal I/R injury can provide a preconditioning effect, through stimulation of the TLR4/NF-kB signaling pathway. Therefore, the subsequent stimulus—the action of IR—does not lead to a substantial increase in the expression of NF-kB.

Angiogenesis and restoration of the tissue microvasculature are well known to play an important role in the restoration of damaged tissues. The analysis of the expression levels of nitric oxide synthase genes (inducible iNOS and endothelial eNOS), which play an important role in maintaining the vascular tone, revealed a significant growth in their level in the group with I/R (Table 6). An increase in iNOS and eNOS in I/R damage is an adaptive response, which results in an augmentation of the level of NO in the blood through the activation of NF-kB/MAPKs signaling pathways [80]. A number of studies have demonstrated the important role of nitric oxide (NO) in reducing vascular thrombosis during the reperfusion period. In addition to that, NO prevents the migration and agglutination of monocytes in blood vessels, affects the tone of the afferent and efferent glomerular arterioles, and is also involved if sodium excretion and the regulation of angiotensin level, which also mediates the regulation of the vascular tone [80,81]. Prior administration of Prx1 or Prx2 was not attended by such a sharp increase in iNOS and eNOS, although their augmentation relative to the control group was registered. As discussed earlier, the decrease in the activation of iNOS and eNOS may be due to the suppression of the NF-kB expression, as well as to the normalization of the microvasculature under the action of exogenous Prx1 and Prx2 [82]. Moreover, it has been shown earlier that exogenous Prx6 administration contributes to the rapid restoration of the blood flow in the microvasculature upon I/R of the small intestine and mesenteric vessels [13,83].

The important role of Prx1 in angiogenesis has been shown quite recently. Addition of the recombinant Prx1 to the culture of mouse vascular endothelial cells provoked an increase in the expression of vascular endothelial growth factor (VEGF), which occurs through the interaction of Prx1 with the TLR4 receptor and subsequent activation of NF-kB and HIF-1a. Conversely, an increase in the HIF-1a factor results in a rise in the VEGF expression and stimulation of vascular growth [84]. Prx1 has been shown to be involved in pulmonary vascularization. Prx1 localized in the fetal lung mesenchyme is involved in cell differentiation and vasculature formation. Transfection of embryonic mesenchymal cells with a genetic construct encoding Prx1 leads to vascular growth [85,86,87]. In addition, mice knockout for the Prx1 gene exhibit defects in the development of vascular smooth muscle cells (VSMC) and impaired rigidity of the extracellular matrix [88]. Thus, Prx1 affects both endothelial cells and vascular smooth muscle cells. An increase in the expression level of Prx1 in VSMC cells increases their proliferative, migratory and invasive activity. Inhibition of TLR4 expression with siRNA in cells with increased Prx1 expression suppresses Prx1-mediated proliferative, migratory, and invasive activity of VSMC, thus supporting the important role of TLR4 in Prx1-mediated inter- and intracellular signaling [76]. Prx2 has also been shown to participate in angiogenesis through the regulation of platelet-derived growth factor (PDGF), which is a potent factor of growth stimulation of endotheliocytes, fibroblasts and smooth muscle cells [89,90]. However, unlike Prx1, Prx2 rather inhibits angiogenesis, since it directly interacts with the PDGFR receptor and thus interferes with the transmission of signal from PDGF [89].

Due to an increase in the ROS/RNS level in renal I/R injury, an increase in the expression level of the transcription factor AP-1, involved in apoptotic cell death [91], was observed. Consequently, along with increased AP-1, an augmentation of the effector caspase-3 levels took place, leading to enhanced apoptotic cell death. Preliminary administration of Prx1 (and less markedly Prx2) prior to I/R injury reduces the level of ROS/RNS in the cells, thereby inhibiting the activation of the ASK-1/JNK/AP-1 signaling pathway and helping to reduce apoptotic death of kidney cells (Table 6, Figure 7).

## 5. Conclusions

Thus, it can be concluded that the introduction of recombinant Prx1 or Prx2 before renal I/R injury favors substantial preservation of the morphological and functional parameters of the kidneys. Importantly, the antioxidant activity of the enzymes is apparently the most important component of the nephroprotective effect, i.e., the higher the peroxidase activity, the better the nephroprotective effect. This observation is confirmed by a more efficient decrease in the level of MDA in the kidney tissue in animals treated with Prx1 and Prx2 (Figure 6). In addition to this, we have shown earlier (in a similar model) that the use of exogenous Prx6 also effectively protects against renal I/R, while the use of a mutant protein having no peroxidase activity does not provide a protective effect [16]. However, a relevant role in the protective effect may also belong to the substrate specificity of exogenous peroxiredoxin. It cannot be excluded that the use of a certain isoform of peroxiredoxin (or a combination of several isoforms), may be effective for various pathologies caused/attended by oxidative stress, depending on the specific type of hydroperoxides formed in the tissues/organs damaged by I/R. It is also important to mention the chaperone activity of Prx1 and Prx2, which provides reduced protein aggregation. This is especially important in glomeruli of the nephrons, because large protein aggregates can reduce their filtration capacity and aggravate I/R injury in the kidney tissue.

We suggest that the use of recombinant peroxiredoxins, in particular Prx1 and Prx2, may be an effective approach for the treatment of renal I/R injury. An important way of application of recombinant peroxiredoxins may be related to their use in perfusion solutions for the preservation isolated kidneys during transplantation. It has been shown that the level of Prx2 in perfusion solution can be an important prognostic factor in cold perfusion of isolated kidneys. The kidney perfusate from living donors had a significantly higher level of Prx2, compared to that from dead donors, allowing the donor kidney to be more “prepared” for reperfusion-mediated oxidative stress and increasing the chances for success of its transplantation [92].

## Figures and Tables

**Table 1:** Oligonucleotides used for real-time PCR. The design of oligonucleotides was carried out with the use of Primer-BLAST (www.ncbi.nlm.nih.gov). The calculated T_m_ for all primers is 61–63 °C. Real-time PCR was performed at T_m_ = 60 °C. PCR products were melted from 60 to 90 °C to assess the specificity of the reaction. In addition amplicon sizes were checked by electrophoresis in 10% PAGE.

Genes	GenBank Accsession #	Oligonucleotides 5′-3′ (F + R)	Amplicon Size, bp
bAct	NM_007393.4	CCTTCCTTCTTGGGTATGGAATCC CACCAGACAGCACTGTGTTGGCA	115
CASP3	NM_009810	AAGGAGCAGCTTTGTGTGTG GAAGAGTTTCGGCTTTCCAG	145
eNOS	NM_021838.2	GAACCTGAGGGTGCCCAG TCCGATTCAACAGTGTCTCCT	71
iNOS	NM_012611.3	GCTACACTTCCAACGCAACA CATGGTGAACACGTTCTTGG	115
IL-6	NM_031168	TAGTCCTTCCTACCCCAATTTCC TTGGTCCTTAGCCACTCCTTC	76
IL-18	NM_008360.1	GTGTTCCAGGACACAACAAG CTTCCTTTTGGCAAGCAAGA	74
NF-kB	NM_008689	CCACGCTCAGCTTGTGAGGGAT GGCCAAGTGCAGAGGTGTCTGAT	106
NRF2	NM_010902	CTCGCTGGAAAAAGAAGTG CCGTCCAGGAGTTCAGAGG	240
KIM-1	NM_001166632.1	TTGCCTTCCGTGTCTCTAAG AGATGTTGTCTTCAGCTCGG	225
CAT	NM_009804	AGCGACCAGATGAAGCAGTG TCCGCTCTCTGTCAAAGTGTG	181
PRDX1	NM_011034	AATGCAAAAATTGGGTATCCTGC CGTGGGACACACAAAAGTAAAGT	149
PRDX2	NM_011563	CACCTGGCGTGGATCAATACC GACCCCTGTAAGCAATGCCC	138
PRDX3	NM_007452	GGTTGCTCGTCATGCAAGTG CCACAGTATGTCTGTCAAACAGG	99
PRDX4	NM_016764	CTCAAACTGACTGACTATCGTGG CGATCCCCAAAAGCGATGATTTC	101
PRDX5	NM_012021	GGCTGTTCTAAGACCCACCTG GGAGCCGAACCTTGCCTTC	154
PRDX6	NM_007453	TAAGGACAGGGACATTTCCATCC CCGTGGAGTTAGGGTAGAGGA	145
SOD1	NM_011434	AACCAGTTGTGTTGTCAGGAC CCACCATGTTTCTTAGAGTGAGG	139
SOD2	NM_013671	GCGGTCGTGTAAACCTCAT CCAGAGCCTCGTGGTACTTC	240
SOD3	NM_011435	CTGAGGACTTCCCAGTGAC GGTGAGGGTGTCAGAGTGT	195

**Table 2:** Changes in the mass of mouse kidneys following 24 and 72 h after I/R and prior administration of recombinant proteins Prx1 and Prx2.

Group of Animals	Change in the Mass of Mouse Kidneys Relative to the Intact Group (%)	
	24 h	72 h
Intact control	100	100
I/R	150 ± 10 *	90 ± 10 *
I/R + Prx1	130 ± 10 *,#	110 ± 10 #
I/R + Prx2	140 ± 10 *	120 ± 10 #

*n* = 30 for each group, *p* ˂ 0.05 relative to the * intact control and # I/R.

**Table 3:** Assessment of morphometric parameters of the kidney tissue after I/R and prior injection of recombinant enzymes Prx1, Prx2 prior to I/R. Histological changes were evaluated by the following scale: (−) normal, (+) slight changes, (++) moderate changes, (+++) significant changes.

Parameter	Intact Control		I/R		I/R + Prx1		I/R + Prx2	
	24 h	72 h	24 h	72 h	24 h	72 h	24 h	72 h
Widening of the Bowman’s capsule	−	−	+	++	+	+	+	++
Congestion of the interstitium	−	−	++	++	++	++	++	++
Interstitial infiltration	−	−	+	+	+	+	+	+
Vessel congestion	−	−	++	++	+	+	++	+
Degeneration of convoluted tubules	−	−	++	+++	+	+	++	+
Degeneration of straight tubules	−	−	+	+	+	-	+	+
Widening of convoluted tubules	−	−	+	+	+	-	+	+
Desquamation of epithelial cells	−	−	++	+++	+	+	++	+
Disruption of epithelial cells	−	−	+++	+++	+	-	+	+

*n* = 10 for each group.

**Table 4:** Urea concentration in the blood of animals over three days after I/R injury of both kidneys.

Group of Animals	Urea Concentration, mg/dL		
	24 h	48 h	72 h
Intact control	57 ± 9	54 ± 7	56 ± 8
I/R	290 ± 40 *	125 ± 10 *	80 ± 10
I/R + Prx1	170 ± 40 *,#	110 ± 20 *	80 ± 10
I/R + Prx2	240 ± 30 *,#	120 ± 20 *	75 ± 10

*n* = 30 for each group, *p* ˂ 0.05 relative to the * intact control and # I/R.

**Table 5:** Creatinine concentration in the blood of mice over three days after I/R injury of both kidneys.

Group of Animals	Creatinine Concentration, mg/dL		
	24 h	48 h	72 h
Intact control	0.28 ± 0.02	0.28 ± 0.02	0.28 ± 0.02
I/R	1.8 ± 0.2 *	1.1 ± 0.3 *	0.7 ± 0.2 *
I/R + Prx1	1.1 ± 0.2 *,#	0.7 ± 0.1 *,#	0.6 ± 0.1 *
I/R + Prx2	1.5 ± 0.2 *,#	0.9 ± 0.2 *	0.7 ± 0.1 *

*n* = 30 for each group, *p* ˂ 0.05 relative to the * intact control and # I/R.

**Table 6:** Changes in the expression level of genes 24 h after I/R injury relative to the intact animals.

Gene	Intact Control	I/R	I/R + Prx1	I/R + Prx2
KIM-1	1.0	106 ± 30 *	71 ± 10 *,#	85 ± 15 *,#
NRF-2	1.0	7.5 ± 0.8 *	0.5 ± 0.2 #	4.0 ± 0.8 *,#
NF-kB	1.0	5.5 ± 0.7 *	1.5 ± 0.3 #	4.0 ± 0.9 *
IL-6	1.0	5.0 ± 0.8 *	2.0 ± 0.5 #	2.5 ± 1.0 #
IL-18	1.0	7.0 ± 1.0 *	0.8 ± 0.2 #	0.8 ± 0.1 #
iNOS	1.0	14 ± 3.0 *	1.3 ± 0.1 #	3.5 ± 0.8 *,#
eNOS	1.0	22.0 ± 4.0 *	2.1 ± 0.5 #	4.0 ± 1.0 *,#
AP-1	1.0	7.5 ± 0.8 *	0.5 ± 0.2 #	4.5 ± 0.2 *,#
Caspase-3	1.0	4.5 ± 0.7 *	0.9 ± 0.3 #	3.5 ± 0.8 *
CAT	1.0	2.5 ± 0.3	1.1 ± 0.2	1.2 ± 0.2
PRDX1	1.0	4.6 ± 0.5 *	1.8 ± 0.2 #	2.2 ± 0.3 *,#
PRDX2	1.0	3.8 ± 0.4 *	1.1 ± 0.2 #	1.3 ± 0.5 #
PRDX3	1.0	2.5 ± 0.3	1.5 ± 0.3	1.6 ± 0.4
PRDX4	1.0	2.2 ± 0.2	1.2 ± 0.2	1.2 ± 0.3
PRDX5	1.0	1.5 ± 0.1	1.3 ± 0.2	1.2 ± 0.2
PRDX6	1.0	4.2 ± 0.3 *	2.0 ± 0.5 #	2.3 ± 0.2 *,#
SOD1	1.0	5.3 ± 0.4 *	1.9 ± 0.3 #	2.1 ± 0.5 #
SOD2	1.0	8.5 ± 1.1 *	2.9 ± 0.4 *,#	3.5 ± 0.4 *,#
SOD3	1.0	2.8 ± 0.3 *	2.0 ± 0.5	2.0 ± 0.4

*n* = 5 for each group, *p* ˂ 0.05 relative to the * intact control and # I/R.
